# Quantifying Population Burden and Effectiveness of Decentralized Surveillance Strategies for Skin-Presenting Neglected Tropical Diseases, Liberia

**DOI:** 10.3201/eid2809.212126

**Published:** 2022-09

**Authors:** Joseph W.S. Timothy, Emerson Rogers, Katherine E. Halliday, Tarnue Mulbah, Michael Marks, Zeela Zaizay, Romeo Giddings, Marie Kempf, Estelle Marion, Stephen L. Walker, Karsor K. Kollie, Rachel L. Pullan

**Affiliations:** London School of Hygiene and Tropical Medicine, London, UK (J.W.S. Timothy, K.E. Halliday, M. Marks, S.L. Walker, R.L. Pullan);; Ministry of Health, Monrovia, Liberia (E. Rogers, T. Mulbah, Z. Zaizay, R. Giddings, K.K. Kollie);; Hospital for Tropical Diseases, London (M. Marks, S.L Walker);; Université d’Angers, Angers, France (M. Kempf, E. Marion);; Centre Hospitalier Universitaire Angers, Angers (M. Kempf)

**Keywords:** neglected tropical diseases, NTDs, skin NTD, population burden, skin, effectiveness, decentralized surveillance strategies, Buruli ulcer, yaws, lymphatic filariasis, leprosy, bacteria, parasites, Liberia

## Abstract

We evaluated programmatic approaches for skin neglected tropical disease (NTD) surveillance and completed a robust estimation of the burden of skin NTDs endemic to West Africa (Buruli ulcer, leprosy, lymphatic filariasis morbidity, and yaws). In Maryland, Liberia, exhaustive case finding by community health workers of 56,285 persons across 92 clusters identified 3,241 suspected cases. A total of 236 skin NTDs (34.0 [95% CI 29.1–38.9]/10,000 persons) were confirmed by midlevel healthcare workers trained using a tailored program. Cases showed a focal and spatially heterogeneous distribution. This community health worker‒led approach showed a higher skin NTD burden than prevailing surveillance mechanisms, but also showed high (95.1%) and equitable population coverage. Specialized training and task-shifting of diagnoses to midlevel health workers led to reliable identification of skin NTDs, but reliability of individual diagnoses varied. This multifaceted evaluation of skin NTD surveillance strategies quantifies benefits and limitations of key approaches promoted by the 2030 NTD roadmap of the World Health Organization.

The World Health Organization (WHO) promotes an integrated strategy for neglected tropical diseases that present primarily with skin changes (skin NTDs) (*1*,*2*). These conditions are characterized by debilitating pathology, chronic disability, and stigma (*2*,*3*). Fundamental challenges for skin NTD programs include a lack of epidemiologic data to determine burden at finer spatial scales and limited guidance on how to sustainably and equitably implement resource-intensive case detection and management interventions within primary healthcare services (*4**‒**7*). This knowledge is essential for progress toward the WHO 2030 roadmap targets that explicitly outline a 10-fold scale-up of skin NTD programs over the next decade (*8*).

Creating and expanding skin NTD programs requires knowledge about disease distribution, particularly co-occurrence of multiple diseases, and subsequent optimization of integrated surveillance strategies at first-line healthcare providers. However, despite clear programmatic need, there are no standardized approaches for estimating prevalence of skin NTDs. Comprehensive, integrated surveys have not yet been evaluated at scale in West Africa, largely because of the epidemiologic traits that characterize skin NTDs: low prevalence, focal distributions, and inaccessibility of affected communities (*4*,*6*,*7*,*9*). This operational gap creates dependence on routine surveillance reports, often considered unreliable because of variable healthcare-seeking behaviors, inadequate diagnostic tools, and unreliable reporting systems (*10*).

Priorities for improving routine surveillance include integrated community-based case finding and midlevel health worker training programs supporting decentralized detection, diagnosis, and case management. The potential for community-based case finding has been demonstrated in Central and West Africa for some diseases, including Buruli ulcer and lymphatic filariasis morbidity (LFM) (*11**–**14*), and recent examples of yaws integration with Buruli ulcer in community outreach programs (*15*). Despite promise, these studies have not rigorously evaluated performance or equity indicators, limiting their broader applicability. WHO recently published a skin NTD diagnostic manual for frontline staff to help improve clinical diagnostic capacity among healthcare workers (*16*). However, the feasibility of training this cadre of healthcare workers to accurately diagnose multiple complex skin conditions has yet to be evaluated.

In light of 2030 skin NTD targets, there is a pressing need to bridge these evidence gaps through operational evaluation (*8*). We aimed to estimate the population-level prevalence of 4 endemic skin NTDs, Buruli ulcer, leprosy, LFM, and yaws, within the routine health infrastructure of Maryland County, Liberia. We implemented community-based case finding and clinical training of midlevel health workers within a stratified 1-stage survey design. We present a detailed breakdown of skin NTD epidemiology and evaluation of integrated surveillance strategies within a programmatic setting.

## Methods

### Study Setting

Maryland County (census population 165,456), a rural county in southeastern Liberia, has the highest levels of absolute poverty (84.0%) in this country (*17*). It is endemic for Buruli ulcer, leprosy, and LFM and borders a yaws-endemic region of Cote d’Ivoire. In March and November 2017, all community health workers (CHWs) and 2 clinicians from each health facility undertook Ministry of Health training modules in recognizing, reporting, and managing skin NTDs, independent from this study.

### Study Design and Participants

We conducted a population-based cluster-randomized cross-sectional survey for Buruli ulcer, leprosy, LFM, and yaws in Maryland County during June‒October 2018 by using a screen and confirm strategy. All communities in the County Health Department of Maryland were eligible for enrollment, and we selected CHW catchment areas as primary sampling units. We combined contiguous CHW catchments that had <300 persons and divided those that had >1,000 persons before selection. We randomly selected 92 clusters (mean population 618) stratified across all 24 health facilities by using probability proportional to size. All residents of selected clusters were eligible and sought for participation in initial screening.

### Ethics

The study protocol was approved by the University of Liberia Institutional Review Board (#18-02-088) and the Ethics Committee of the London School of Hygiene and Tropical Medicine (#14698). Community meetings were held in all study clusters before implementation. We obtained verbal consent from adult residents for household participation in screening, and written consent from adults, or guardians if persons were <18 years of age, for quality control and case verification visits. All skin NTDs and other diagnosed skin conditions were immediately referred for treatment at health facilities in line with national guidelines. This study is registered with ClinicalTrials.gov (https://www.clinicaltrials.gov), no. NCT03683745.

### Procedures

We conducted an exhaustive population screening in selected CHW catchment areas (Appendix). CHWs visited all households within their catchment communities over a 5-day period, completed a simple census, and screened residents for suspected skin NTDs on the basis of interviewee report, using photographs of clinical manifestations. The household head or primary caregiver were directly prompted to act as a proxy respondent for absent members. Visited households were provided with quick response‒coded study identification cards, and persons who had suspected cases were provided a separate individually identifiable identification card.

One week after community screening, suspected case lists were provided to mobile verification teams for home-based follow-up, diagnosis, and referral. Before survey activities, a team of 7 nationally recruited midlevel health workers (physician assistants) attended a 5-day training course on diagnosis and management of skin NTDs held at a national referral center for Buruli ulcer and leprosy in Ganta, Nimba County, and led by Ministry of Health NTD program (E.R. and T.M.) and UK-based experts, including a consultant tropical dermatologist (M.M., S.L.W, and J.W.S.T.). During household visits, trained skin NTD verifiers performed detailed clinical examination of all suspected persons who had cases before diagnosis.

All survey stages were evaluated through separate quality control (QC) surveys. CHW screening was evaluated by an independent community health services supervisor (CHSS), who randomly visited 10–15 households/cluster during the week after CHW screening activities. At each household, study identification cards were recaptured and household information was collected. The CHSS performed skin examinations of all consenting household members and recorded all skin lesions comparable to the photographic case definitions used by CHWs. Clinical diagnoses were validated in a purposively selected subpopulation of verified cases by clinically trained members of the national NTD program (E.R., T.M., and R.G). Additional QC was implemented through deployment of global positioning system‒enabled electronic data collection devices running open data kit‒based data collection platforms across all survey stages.

### Outcomes

The primary outcome was prevalence of all skin NTDs diagnosed by trained verification teams. We confirmed clinically suspected Buruli ulcer by using an IS2404 PCR with swab specimens or fine-needle aspirates (*18*). We defined yaws cases as a clinically suspicious lesion plus dual serologic positivity by using a syphilis dual path platform lateral flow assay for both treponemal and nontreponemal antibodies (ChemBio, https://chembio.com). All serologically confirmed yaws cases also underwent PCR confirmation (tp47) of lesion swab specimens. We based LFM and leprosy diagnoses on clinical assessment of signs and symptoms.

We also collected routine program data from Maryland County aggregated by the county health office on all skin NTD outcomes from the year before survey implementation. All diagnoses through the routine program were made on the basis of clinical assessment. We compared the annual new case detection rate to the prevalence of survey cases that we confirmed as being previously unknown to the health system. During verification, a case-patient was determined as unknown to the health system by interviewing the patient and CHSS and by cross-checking all survey cases against county records.

### Sample Size and Statistical Analysis

We performed data management and statistical analyses by using R version 4.0.1 (https://www.r-project.org). We assumed a population-level skin NTD prevalence of 5 cases/10,000 persons, absolute precision of 3.5 cases/10,000 persons, a design effect of 3.5, a participation rate of 0.8, and a finite population correction factor. The required sample size was 48,478 by using standard formulas. We estimated prevalence through design-based inference as a stratified 1-stage cluster design with variance estimated by using Jackknife Repeated Replication Survey version 3.36, (https://am.air.org/Manual/Tools/Variance-Estimation/Jackknife-Repeated-Replication). We estimated intraclass correlation coefficients (ICC) from intercept-only binomial mixed effects models (lme4 version 1.1–23) (*19*). We analyzed operational factors associated with survey participation by using binomial mixed effect and conditional logistic regression (survival version 3.1–12, https://rdrr.io/cran/survival/man/clogit.html) with model-building approaches (Appendix). We used the Cohen κ and crude agreement to estimate interrater reliability of all clinical diagnoses (psych version 1.9.12, https://cran.r-project.org/web/packages/psych/psych.pdf).

## Results

**Figure 1:**
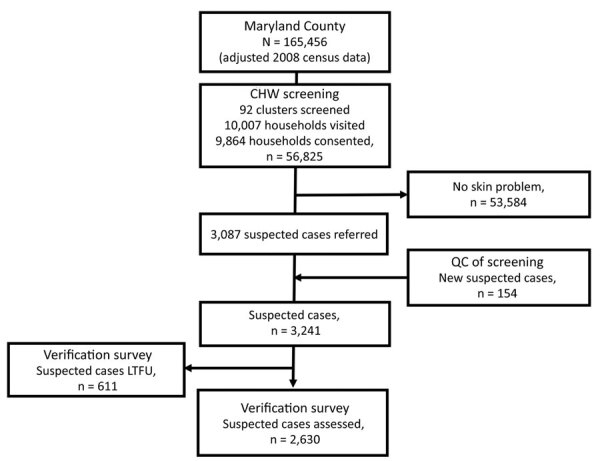
Study population flowchart for study quantify population burden and effectiveness of decentralized surveillance strategies for skin-presenting neglected tropical diseases, Maryland County, Liberia. Consort diagram shows selection, screening, quality control, and verification stages. CHW, community health worker; LFTU, lost to follow-up (did not continue to participate in follow-up contacts); QC, quality control.

We visited 10,007 households across 92 clusters (143 refused, 1.4%) and included 56,825 persons (49.8% female, 47.3% <18 years of age) in the sample population (Figure 1). In total, 34,916 persons were present during CHW household screening visits to observe photographs of skin NTDs. The remaining 38.6% were absent at the time of survey, and referrals among this group were based on proxy responses.

Among the sample population, 3,087 persons (5.4%, 95% CI 5.2–5.6) were referred by CHWs because these persons had possible skin NTD symptoms. Median age of referrals was 27 years (35.7% female, increasing to 48.3% when excluding hydrocoele; 102 missing age or sex data). We observed a linear increase in referral rates by age (p<0.0001), with an approximate threshold at 35 years, over which referrals increased more than 2-fold from 4.1/100 persons screened (95% CI 3.9–4.3) to 8.6/100 persons screened (95% CI 8.1–9.1). CHW referral rates varied substantially by cluster (range 0.5–23.0/100 persons screened; ICC 0.11) and health district (3.1–7.0/100 persons screened; ICC 0.01). Models exploring associations between referral rates and potential operationally relevant variables indicated only older CHW age to be associated with reduced odds of referral (>35 years of age; odds ratio 0.59, 95% CI 0.43–0.81; p = 0.001) (Appendix).

**Figure 2:**
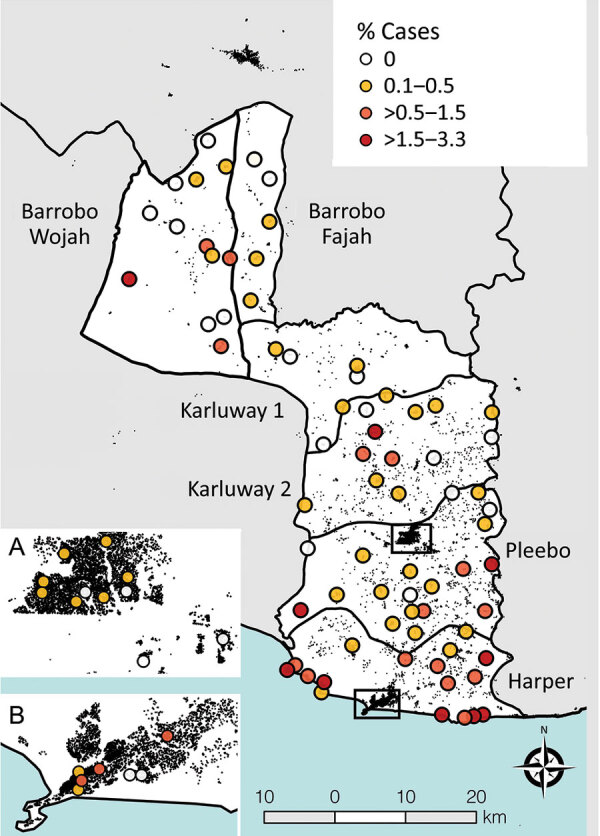
Cluster-level prevalence of all skin-presenting neglected tropical diseases combined, Maryland County, Liberia, June‒October 2018. Inset boxes show major urban areas Pleebo (A) and Harper (B). Black features are buildings (OpenStreetMap contributors) to highlight increasing rurality in northern districts.

Mobile verification teams successfully followed up with 2,630 case-patients (81.1% of those referred). This group had minor differences in age compared with those who could not be found for follow-up (27.7 years [95% CI 26.1–29.3 years] vs. 30.3 years [95% CI 29.4–31.1]) but no overt difference in sex (35.0% [95% CI 31.0%–39.1%] female patients followed up vs. 36.7% [95% CI 34.8%–38.6%] female patients not followed up) or implementation district of residence (p = 0.15). We diagnosed 236 cases of skin NTDs (Table 1), a crude prevalence of 41.5 skin NTDs/10,000 persons and a design-adjusted prevalence of 34.0 (95% CI 29.1–38.9) skin NTDs/10,000 persons (Figure 2). The most prevalent condition was LFM, causing 111 lymphedema (17.5 [95% CI 14.1–21.0] cases/10,000 persons) and 58 hydrocoele cases (8.5 [95% CI 4.8–12.3] cases/10,000 persons). We identified 55 cases of suspected Buruli ulcer on the basis of clinical case definitions, although only 4 were confirmed by PCR (0.9 [95% CI 0–1.9] cases/10,000 persons), establishing PCR-confirmed Buruli ulcer as the rarest outcome (Appendix).

**Table 1:** Final prevalence estimates of primary and secondary skin-NTD outcomes, Liberia*

Disease	Total no. cases	Crude prevalence/10,000 persons (95% CI)	Design-adjusted population prevalence/10,000 persons (95% CI)	Median age, y	Female, %	Cluster prevalence range/10,000 persons	ICC†
All skin NTDs	236	41.5 (36.2–46.8)	34.0 (29.1–38.9)	42	42.3	0‒330	0.27
Buruli ulcer	4	0.7 (0.1–1.4)	0.9 (0–1.8)	16.5	50.0	0–39.4	NA
Leprosy	39	6.9 (4.7–9.0)	4.4 (3.3–5.5)	44	42.8	0–74.1	0.18
LF lymphedema	111	19.5 (15.9–23.2)	17.5 (14.1–21.0)	48	67.3	0–209.7	0.41
LF hydrocele	58	10.2 (7.6–12.8)	8.5 (4.8–12.3)	43	0	0–256.4	0.43
Active yaws	24	4.2 (2.5–5.9)	2.6 (1.4–3.9)	10	25.0	0‒205	0.93

*Age and sex data were missing for 9 skin NTD cases. ICC, intraclass correlation coefficient; LF, lymphatic filariasis; NA, not available; NTD, neglected tropical disease. †Not estimated for Buruli ulcer.

**Figure 3:**
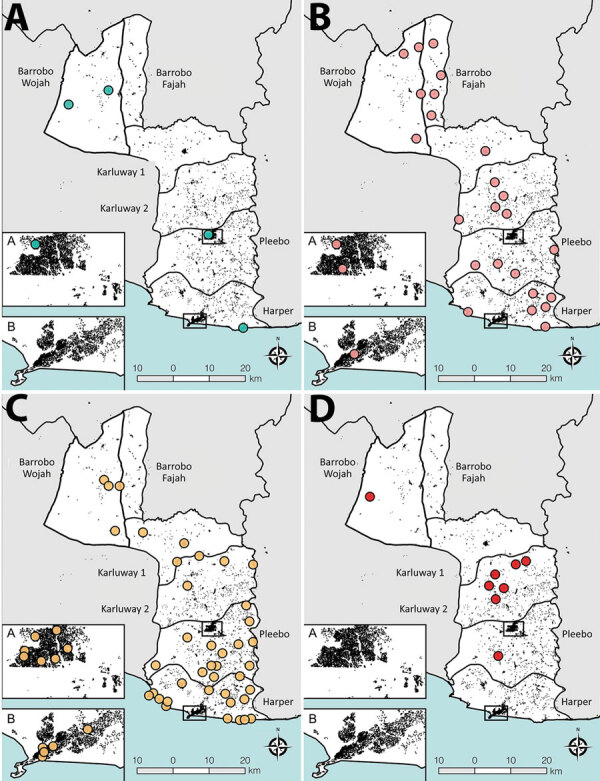
Spatial distribution and occurrence of skin-presenting neglected tropical diseases , Maryland County, Liberia, June‒October 2018. A) Buruli ulcer, B) leprosy, C) lymphatic filariasis morbidity; D) yaws. Points represent cluster centroids and not absolute location of cases.

**Figure 4:**
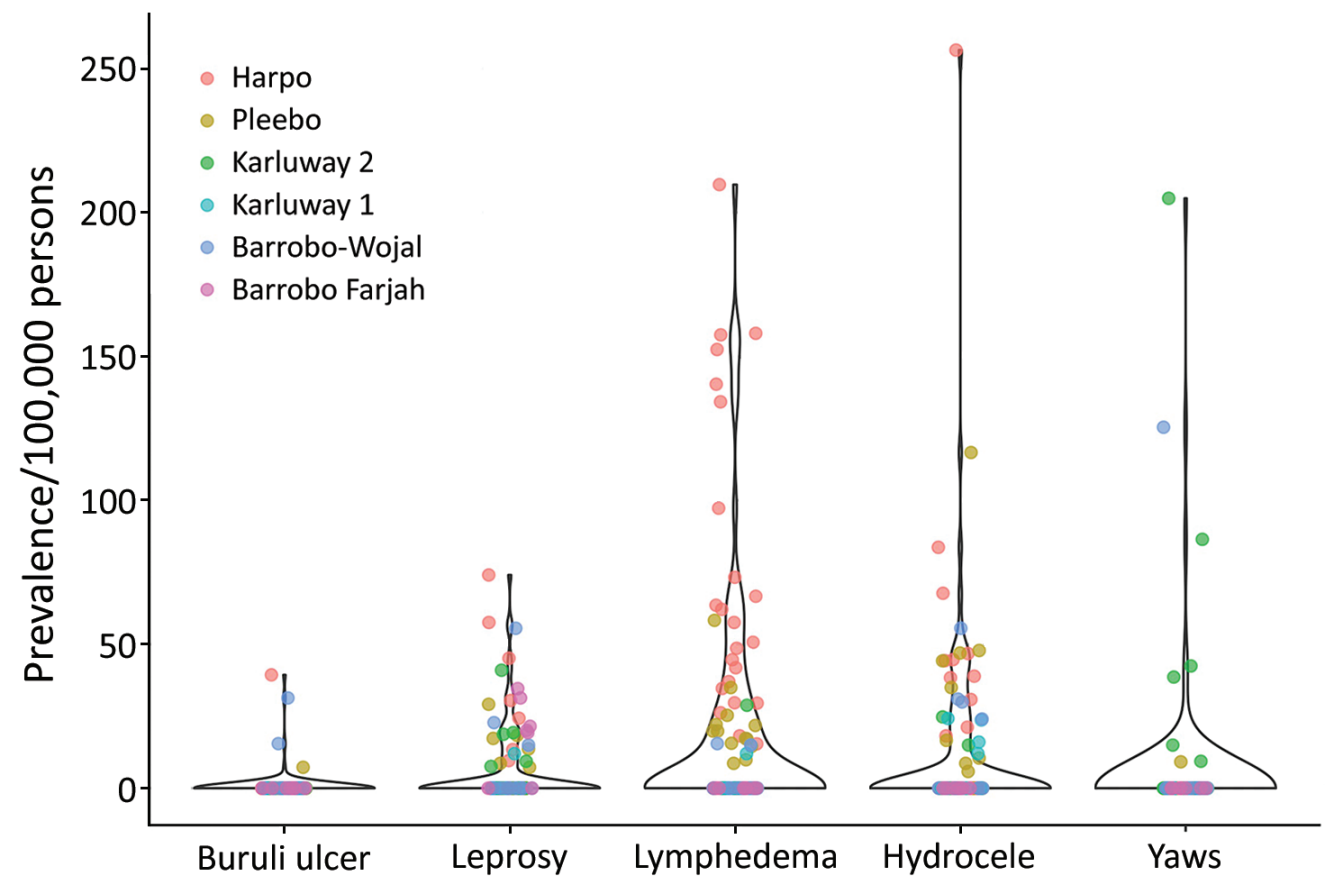
Cluster-level prevalence of skin-presenting neglected tropical diseases, Maryland County, Liberia, June‒October 2018. Colors denote health district of cluster.

Prevalence of any skin NTD was focally distributed within communities (ICC 0.27), with considerable heterogeneity between clusters (range 0–330 case/10,000 persons) (Figures 3,4). Analysis of individual skin NTDs showed a greater degree of spatial heterogeneity, with LFM and yaws exhibiting particularly focal distributions (Table 1). Few clusters were co-endemic for more than 1 skin NTD (22 of 92, 23.9%) and only 1 cluster was co-endemic for >2 diseases. Of potential cases identified in screening, 91.0% (2,394/2,630) were diagnosed with conditions not included within the primary outcome, including superficial fungal infections (471 cases, 17.9% of verified cases), scabies (316 cases, 12.0%), scrotal hernia (279 cases, 10.6%) and skin ulcers of unknown etiology (110 cases, 4.2%) (Appendix).

**Figure 5:**
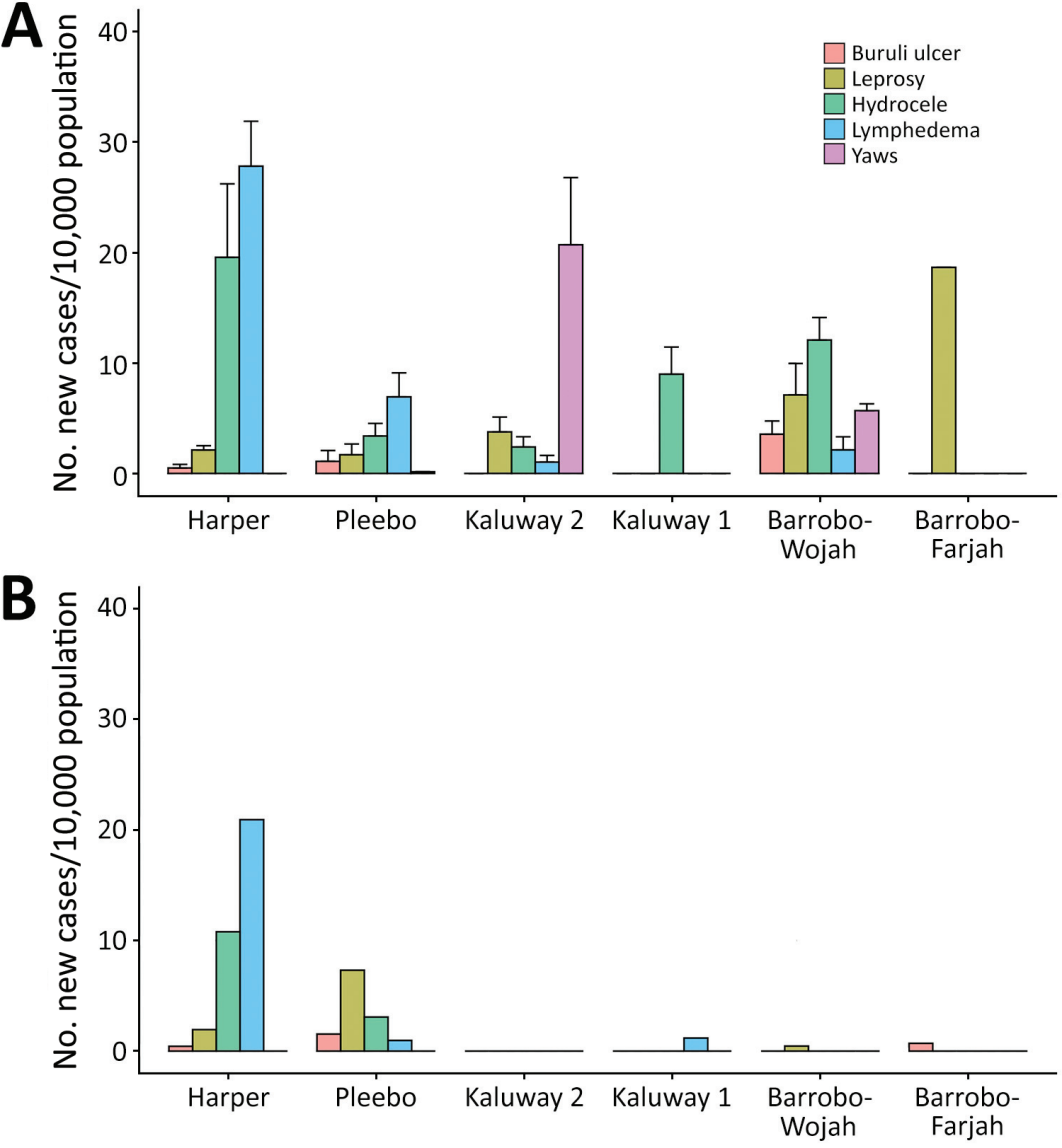
Comparison of cases of skin-presenting neglected tropical diseases before and after survey Maryland County, Liberia. A) Survey cases previously unknown to the health system; B) annual new case detection rates from routine health system records extracted from the 12 months before survey implementation. Note that plots are comparing point prevalence (A) with annual new case detection rates (B). Routine diagnosis is limited to clinical suspicion for Buruli ulcer. If survey estimates are extended to include all clinically suspected cases of Buruli ulcer, we estimate a countrywide prevalence of 32.4 (95% CI 27.4–37.3) previously unknown cases/10,000 persons.

The new case detection rate from existing county-level health records in 2017 was 13.8 cases/10,000 persons compared with our survey point prevalence estimate of 25.4 (95% CI 21.3–29.5) previously unidentified cases/10,000 persons (Figure 5). Overall, there was no evidence of differences in age and sex of case-patients detected through routine reporting. Among leprosy case-patients only, those we detected by using survey methods were older (46.3 vs. 35.2 years; p = 0.02), and there was a greater proportion of paucibacillary leprosy relative to routine data (53.8% vs. 22.9%; p = 0.006).

To assess performance of CHW screening, QC surveys were conducted in 1,382 randomly sampled households (1,379 consented, 99.8%) in 91 clusters before verification of cases took place. Among the QC sample population, 95.1% of households (1,320 of 1,379) reported being visited by the local CHW and shown skin NTD photographs, with no evidence of socioeconomic disparities between households visited or missed (Appendix).

QC teams enumerated 8,021 persons and performed skin examinations on 4,268 household members (53.2%) among 4,409 approached (141 refused, 3.2%). Among persons examined, clinical field officers (trained CHSS cohort) identified 503 cases (11.8 [95% CI 10.8–12.8] cases/100 examined) of skin lesions similar in appearance to photograph-based CHW case definitions. Among the 503 patients who had skin lesions, clinical field officers recaptured patient identification cards from 349 to estimate sensitivity of screening (69.4%; CHSS new case detection rate 3.6 [95% CI 3.1–4.2] cases/100 persons). There was good concordance with CHW referrals for age and proportion of female referrals. We also conducted a sensitivity analysis of the effect of reduced sensitivity on prevalence estimates (Appendix).

We assessed the reliability of clinical diagnoses made by verification teams through separate follow-up QC surveys immediately after case verification activities. We reached 174 of 2,630 verified cases (6.6%) across 16 health facilities and 36 clusters. The crude agreement of all 174 diagnoses as skin NTD was 82.8% (Cohen κ 0.69, 95% CI 0.59–0.79), indicating substantial agreement between raters. Excluding other skin diseases, crude agreement (62.0%) and Cohen κ (0.51, 95% CI 0.39–0.64) were lower for skin NTDs, with a tendency for overdiagnosis among verification teams (Table 2). For individual skin NTDs, we did not estimate Cohen κ because of high prevalence index introduced by our sampling approach (*20*), but crude agreement between raters showed considerable variation between diseases (Table 2).

**Table 2:** Summary of interrater reliability scores of skin-NTD clinical diagnoses, Liberia*

Disease	Total survey cases assessed	Total QC clinical diagnoses	Agreement	Verifier-only diagnoses	QC-only diagnosis	Agreement, %	Alternative diagnoses
Suspected Buruli ulcer	15	11	10	5	1	62.5	Traumatic ulcer, tropical ulcer
Leprosy	12	7	7	5	0	58.3	Vitiligo, tinea corporis
LF lymphedema	25	27	24	1	3	85.7	Non-LF edema
LF hydrocele	17	14	8	9	6	34.8	Hernia, non-LF hydrocele
Other skin disease	105	115	95	10	20	76.0	None
Combined skin NTDs	69	59	49	20	10	62.0†	None

*Overall agreement; 82.8%; Cohen κ, all outcomes: 0.69 (95% CI 0.59–0.79). LF, lymphatic filariasis; NTD, neglected tropical disease; QC, quality control. †Cohen κ: 0.51 (0.39‒0.64).

## Discussion

This study was a programmatic-scale integrated skin NTD prevalence survey in West Africa and was conducted entirely within the routine health infrastructure of Maryland County, Liberia. Our results show that skin NTDs in this setting are underreported, spatially heterogeneous, and highly focal, imparting a considerable unmet burden on this largely rural and periurban population.

We concurrently provide new evidence on the effectiveness of surveillance strategies that form the basis of skin NTD program delivery outlined in the WHO 2030 NTD roadmap (*21*). We demonstrate that large-scale screening by CHWs can find unreported cases of stigmatizing diseases while achieving high and equitable coverage among hard-to-reach communities. We also quantified major limitations in sensitivity and specificity from using our chosen approach with this workforce. Integrated clinical training of nonphysician healthcare workers facilitated reliable differentiation between any skin NTD and other skin conditions reported by participants. However, reliability of disease-specific diagnoses of skin NTDs was variable.

The greatest disease burden in Maryland County was attributable to LFM, with both BU and yaws showed markedly lower prevalence. Burden across all skin NTDs was higher than reported through routine surveillance systems for the county, as well as those typically reported in surveillance records nationally and across other co-endemic states in West Africa, although Buruli ulcer remains comparable if limited to PCR-confirmed cases (*5*,*7*,*22*). All diseases appeared spatially heterogeneous in occurrence and prevalence at this implementation scale. The explanatory factors underlying these observations are probably multifaceted, given diverse transmission dynamics, a combination of climatic, ecologic, and sociodemographic (*23**–**26*). However, given highly focal distributions, these observations could be attributable to sampling error.

Population-level skin NTD surveys have previously been undertaken in Ethiopia, Rwanda, and Cameroon (*12**,**27**–**28*), demonstrating a similarly high unmet burden. We believe the additional granularity and operational evaluation in our study provides additional strong justification for integrated approaches to skin NTD surveillance. We demonstrated that at the cluster level, most communities did not have individual skin NTDs, resulting in wasted resources if using nonintegrated surveillance strategies. Although findings indicate that disease-specific interventions could be targeted to smaller implementation units, sampling effort required for accurate delineation might outweigh benefits of microplanning.

The use of CHWs for disease-specific surveillance is common in West Africa, particularly for Buruli ulcer, for which increased case numbers or earlier stages of detection have been reported in quasiexperimental studies (*13*,*29*,*30*). Our findings illustrate the feasibility of training a rural community-based workforce with limited smartphone experience to screen for multiple diseases, reliably capture electronic data, and achieve high and equitable population coverage. CHWs identified a large proportion of previously undetected cases, even in a setting with recent previous training of CHWs and formal health workers. We also found no evidence households missing during screening were systematically omitted on the basis of socioeconomic indicators. However, we observed and quantified the probable underestimation of referable skin lesion burden by using our chosen approach. In addition, 91% of persons with verified cases were ultimately diagnosed with non–skin NTD etiology, including a large number of communicable skin diseases (corroborating recent dermatologic surveys in neighboring Côte d’Ivoire [(*31*)]), debilitating ulcers, and scrotal hernias. Given widespread use of CHWs for skin NTD surveillance, our results quantify major considerations with this approach, including management of a potentially large additional burden of disease.

Sustainable skin NTD programs also depend on decentralized diagnosis and case management by mid-level health workers. The performance of integrated skin NTD training programs has not been formally evaluated, despite recent WHO publication of a manual for frontline healthcare workers (*16*). Our findings show that a tailored training program reliably identified skin NTDs but that agreement on specific diagnoses could be inconsistent, particularly in the case of hydrocele and leprosy. Furthermore, confirmation rates of clinically suspected Buruli ulcer and yaws highlight the need for laboratory support for diagnosis. Previous studies in West Africa showed success in developing clinical algorithms for common skin diseases (*32*), and research continues on alternative algorithmic or telemedicine approaches to support decentralized clinical decision-making (*33*,*34*). Our findings support the need for further evaluation of integrated training programs to support frontline healthcare workers, especially in settings in which laboratory support is limited.

The first limitation of our study was that we relied on CHWs to conduct screening, a strategy that might have led to us miss the most marginalized households at higher risk for skin NTDs. Nevertheless, our QC survey suggested high coverage, a finding also supported when cross-comparing household global positioning system points with satellite imagery. Second, screening relied upon self-report and proxy-report of stigmatized conditions. We quantified a degree of loss in sensitivity through QC skin examinations, but inclusion might have been further biased downwards if affected persons were less willing to participate. Third, ascertainment of leprosy and LFM was dependent on clinical diagnosis, with variable reliability potentially biasing estimates from true population prevalence. Finally, we observed a notable percentage (≈19%) of patients who did not continue follow-up between screening and verification stages, although we did not see overt differences in the demographics of the censored population. We would expect this aspect to bias final prevalence estimates down, but the magnitude of this effect remains unclear.

With the new WHO 2030 NTD roadmap explicitly mapping out a 10-fold scale-up of skin NTD programs, there is an urgent need to better clarify disease burden and strategies for integrated surveillance to support this global transition (*8*). Our results provide a multifaceted overview of disease epidemiology and operational evaluation of surveillance strategies that can guide countries who are beginning integrated skin NTD programs.

Appendix Additional information on quantifying population burden and effectiveness of decentralized surveillance strategies for skin-presenting neglected tropical diseases, Liberia.
