# Supplementary material for: Quantifying Population Burden and Effectiveness of Decentralized Surveillance Strategies for Skin-Presenting Neglected Tropical Diseases, Liberia
Source: Emerg Infect Dis. 2022 Sep;28(9):1755–64. doi: 10.3201/eid2809.212126 (PMC9423900; doi:10.3201/eid2809.212126)
Supplement: Appendix — Additional information on quantifying population burden and effectiveness of decentralized surveillance strategies for skin-presenting neglected tropical diseases, Liberia. [file 21-2126-Techapp-s1.pdf]

Article DOI: https://doi.org/10.3201/eid2809.212126

Quantifying Population Burden and Effectiveness of Decentralized Surveillance Strategies for Skin-Presenting Neglected Tropical Diseases, Liberia

Appendix

Methods

CHW Training and Data Collection

CHWs were trained at health district level during implementation to perform a full census and screen their resident community for visible signs of skin NTDs using photographs of common clinical presentations. The 3-day course was led by survey verification teams who cascaded the training program, originally delivered via training of trainers by MoH NTD program staff and LSHTM collaborators. Community screening commenced 1-day after the completion of training to enable CHWs to return to their communities.

All data collected by CHWs was acquired electronically by using smartphones. Due to variable rates of literacy and experience with smartphones, the quantity of data collection was limited during CHW screening. We trained CHWs to collect GPS locations for every household visited and household demographic variables including the number of persons who slept in the household the previous night. We did not collect individual information for all household members due to CHW capacity and time constraints. For this reason, we relied on age-standardized estimates to make age-related estimates of CHW data among the screened population using United Nations 2015 national population pyramids (*1*). The process to identify suspected cases involved gathering all present members of the household to a well-lit area and showing 12 pages of A4 laminated photographs with all common presentations of skin NTDs. We showed a comprehensive range of visible presentations to try and capture as much of the community skin NTD burden, including, early-stage lesions. The photos included were reviewed

by international skin NTD experts and UK-based consultants in tropical dermatology and infectious diseases and the final tool included presentations of the following:

Buruli ulcer nodules

Buruli ulcer plaques

Buruli ulcer edema of the limbs (arms and legs) and face

Buruli ulcer osteomyelitis involving pustular discharge

Ulcerative forms of Buruli ulcer (category I, category II and category III)

Single hypopigmented skin patches (pale and reddish) on dark skin

Multiple hypopigmented skin patches (pale and reddish) on dark skin

Lepromatous leprosy (leonine facies with madarosis, infiltrated nodules on face and ears, diffuse nodules on the trunk and limbs)

Leprosy-related deformities (resorption of fingers and toes)

Filarial lymphedema of the limbs (arm and leg)

Hydrocele

Yaws ulcers (crusted and open)

Yaws papilloma (single and multiple)

We excluded secondary yaws from our clinical case definitions due to its nonspecific presentations.

CHWs were trained to show the photos to all members of the household. At the end of showing the photos the CHW would ask the household if they or anyone in the household has a skin problem that "looks like any of the photos." If no household members absent at the time of the visit were initially referred by proxy, the household head or primary caregiver were directly prompted to act as the proxy respondent. Individual information was collected at this stage for all suspected skin NTD cases (age, sex, lesion type and phone number) and each unique patient provided with a QR-coded patient ID card. Follow-up teams would re-capture patient ID cards to ensure accurate patient linkage between survey stages.

All data collection tools were designed on an ODK-based platform (SurveyCTO, Dobility, USA) with data checks and audits steps built within the form to ensure data reliability. Data collection devices were android-based smartphones that costs $35 per unit (Tecno Rise 32). Among the CHW cohort, only 18 of 94 CHWs (19.1%) owned a smartphone with 26 (27.7%) not owning any type of phone and 54 (57.5%) had not completed secondary school. At every household, CHWs were instructed to scan and distribute household ID cards. We also monitored coverage using GPS coordinates collected at each household. Upon completion of surveys, CHW data was uploaded and building coverage was checked against most recently available open-source satellite imagery. If low building coverage was observed, CHW were asked to return complete the missing areas. This validation step was not possible in Barrobo districts due to a combination of heavy rainfall and poor mobile network coverage. Due to limited power networks given the rural location of most clusters, CHWs were also provided with high-capacity power banks to enable this work (48,000 mAh), as screening lasted for 7 days.

Midlevel Health Worker Training Program

We recruited clinically trained verifiers for the duration of Maryland survey activities (4-months). All verifiers required physician assistant qualification with clinical experience as a mid-level health worker in Liberia. Our verifier cohort included nurses, physician assistants, community health services supervisors and officers in charge. Following selection, verification teams were trained on the diagnosis of skin NTDs using a novel integrated 5-day training workshop of clinical dermatology led by the Ministry of Health NTD program (ER, TM) and UK-based experts including a consultant tropical dermatologist (MM, SW,JT). The residential training was based at Ganta Rehab Centre in Nimba County, a national referral hospital for Buruli ulcer and leprosy. Additional patients with lymphatic filariais were recruited from nearby communities to facilitate practical experience. No symptomatic yaws patients were available for the training program.

Training was initiated with an introductory day of fundamental dermatological concepts and common skin diseases. Common skin disease were based on local epidemiology and focused on superficial fungal infections, impetigo and scabies, scrotal hernia and ulcers of alternative etiology. Pedagogic elements were followed by a half day skin clinic based in a nearby village to enable trainees experience with common presentations and differential diagnoses. The common skin disease module was followed by pedagogic and practical training with skin NTD patients

over the remaining 3.5 days. Due to a lack of validated clinical algorithms we did not train verification teams using an algorithm-based approach for diagnosis of skin NTDs. We instead used a global assessment of symptoms and provided job aids with common clinical symptoms and epidemiologic characteristics of diseases. Pedagogic and practical training elements were aligned with these definitions throughout training and job aids were able to provide decision-making support in the field. The training program finished with a clinical assessment from cases recruited in the community and written exam – with clinical feedback provided by the program's lead dermatologist. Additional training on the use of electronic data collection tools was undertaken over 2 days in Maryland County before initiation of activities (ER, JT, KEH).

Disease Verification

Following CHW screening all cases were followed-up by verification teams trained on the MLHW training program. One member of the verification team was assigned to a health facility and provided with a full line list of suspected cases. Team members were based in the community for 7–10 days to follow-up all patients and coordinated activities with CHWs and the community health services supervisor (CHSS) of the facility. Data was captured on electronic devices with custom-made ODK-based surveys including assistive protocols for diagnostic approaches (skin examinations, swab sampling, rapid diagnostic tests). All laboratory samples were stored in cell lysis solution (Catalog no. 158908; QIAGEN, https://www.qiagen.com) transported in vaccine carriers and stored daily in facility freezers. Samples were transported to a −20°C central freezer at JJ Dossen Hospital, Harper after each phase of verification before shipment to the London School of Hygiene and Tropical Medicine, United Kingdom.

Quality Control of Screening Process

We aimed to perform quality control of the CHW screening process in all survey clusters. QC surveys were performed at household level to assess 1) coverage of CHW screening, 2) sensitivity of photo-based screening by CHWs, and 3) sociodemographic factors systematically associated with exclusion from CHW screening. Quality control surveys were undertaken by the CHSS of each of the county's 24 health facilities between 1–6 days after CHWs completed screening. Training of the CHSS was delivered by members of the verification team who participated in a 2-day training-of-trainers program led by members of MoH and LSHTM research team (ER, JT, KEH). Each CHSS was trained one-to-one for a full day by a member of the verification team and the first day of QC surveys individually supervised. The CHSS visited

each cluster for one full day resulting in 3–4 days of surveys per facility. Each CHSS was provided with an electronic data capture tool that defined a random start point in the village and different random walk procedure each day. At each household, the CHSS would record household-level information to validate CHW information and collect additional sociodemographic data. Present household members were assembled and asked to verbally report if the CHW visited to show skin NTD photos and if so, requested the household ID for re-capture. Each consenting individual subsequently underwent a full body skin examination in a private setting with appropriate lighting. Each CHSS was trained to record lesions that looked visually similar to those in photo-based screening tools used by CHWs. All patients with lesions identified were asked to present their individual ID which was then re-captured if available to differentiate between new cases and those previously identified during CHWs screening.

Quality Control of Verification Process

We estimated the reliability of clinical skin NTD diagnoses made by verification teams using QC surveys. After completion of verification, QC teams visited persons who could be reached from the health facility on the same day of visit due to logistical constraints. From the patients within this defined area we targeted all patients diagnosed with skin NTDs by verification teams and a random selection of patients with alternative diagnoses. QC of clinical diagnoses was made by members of the national case management NTD control program (ER, RG, TM) visiting patients in their own home. No cases were assessed in either Barrobo district as the area became inaccessible due to adverse weather conditions. Clinical diagnoses of yaws were not were not subject to analysis as cases were treated with azithromycin and follow-up visits could be over 14-days from initial diagnosis. We used inter-rater reliability measures to compare clinical diagnoses with measures of kappa score for all diagnoses (R, psych v1.9.12). For individual skin NTD outcomes kappa scores were not appropriate measures due to high prevalence index introduced by the sampling design (*2*), and we instead present crude agreement measures.

Statistical Analysis (Survey Analysis and Modeling)

To estimate population prevalence of skin NTDs, prevalence was estimated to account for a stratified design with primary sampling units (PSU) selected within health facility strata proportional to size. Prevalence estimates and variance were adjusted for both strata population and first order inclusion probabilities of PSUs. The survey sampling frame used adjusted 2008

census population data to ensure common implementation with Maryland County health team. Due to inaccuracies in population census data, some cluster boundaries were not aligned with CHW catchments and some populations were evidently inaccurate based on CHW survey data coupled with satellite imagery analysis. To account for this, if survey clusters were under minimum sizes, the nearest contiguous cluster(s) from the original sampling frame was also screened by the same CHW, with both then included as a single survey cluster. If cluster boundaries did not match true CHW catchments, cluster boundaries were re-drawn. To account for these changes during analysis of prevalence estimates, strata cluster numbers and cluster-level inclusion probabilities were re-calculated based on updated boundaries. This resulted in a sample of 92 from 185 total primary sampling units.

To assess the equity of CHW screening process, we used a matched case-control approach to identify household-level sociodemographic information that was systematically associated with exclusion from CHW screening. Cases were defined as households not visited by CHWs during screening, confirmed both verbally and by the absence of a QR-coded household ID card. We aimed to randomly selected 4 control households per case from within the same cluster. For some households, it was only possible to select 2–3 matched controls due to limited numbers. We built a conditional logistic regression model using sociodemographic data collected by QC teams analyzed in R (survival version 3.1–12). All independent variables were tested for univariate association using likelihood ratio tests against initial parameter estimates. All variables showing a statistical association below a p-value threshold of 0.20 were included in a final multivariable model. Quantitative variables were assessed using pre-defined categories and included as linear predictors if categories did not improve model fit at a pre-defined threshold of $p = 0.05$ (likelihood ratio test).

To understand CHW characteristics that could explain observed heterogeneity in referral rates between clusters, we used a mixed-effects generalized linear modeling approach (binomial family distribution). We defined the binary outcome at household level, with a positive outcome as the household having at least one individual referred for second stage verification. We collected additional sociodemographic information from all CHWs during training workshops for screening activities as potential exposures at cluster level affecting referral rates. Additional cluster-level variables were extracted from open-source geographic information system datasets to define clusters as rural or urban, and the distance of the cluster to stable night lights

(WorldPop, www.worldpop.org). A baseline model was set that included the number of persons and sex distribution at household level and two proxy measure of urbanization. Cluster-level (CHW-level) variables were added for univariate analysis and all variables included in a final model with a random intercept assigned to cluster-level covariates. Quantitative variables were assessed using pre-defined categories and included as linear predictors if categories did not improve model fit at a pre-defined threshold of p = 0.05 (likelihood ratio test).

Results

Community Health Worker Screening Results

We quantified the proportion of household members who saw photos during CHW screening, with 34,916 of 56,825 persons recorded as present during screening surveys (61.4%). The remaining 38.6% not present during surveys, therefore, relied on proxy answers from household members for referral. We present the distributions of the number of household members versus the number present to see photos in Appendix Figure 1.

The CHW screening process identified 3,087 persons who verbally reported the presence of skin NTD symptoms. There was operationally relevant variation in referral rate of all skin NTDs observed at both health district (range 3.1% - 7.0%) and cluster level (range 0.5 - 23.0), which are shown in Appendix Table 1 and Appendix Figure 2. Appendix Table 1 also quantifies the high referral rates in districts with large peri-urban centers (Harper and Pleebo). While epidemiologic and environmental differences may drive natural variation in skin disease at these spatial scales, CHW demographics also varied by health district. For example, Harper and Pleebo contained 74.2% of all CHWs with secondary school qualifications despite only 56 of 92 (60.1%) of CHWs operating in these districts. We therefore aimed to identify possible factors within our CHW cohort associated with different rates of referral through a hierarchical modeling approach (Appendix Table 2). We observed an independent, inverse association between the age of the cluster's CHW (OR 0.59, 95% CI 0.43 – 0.81 p = 0.001) and the odds of a household being referred during screening. We also observed weak inverse association between CHW education level and odds of referral (Secondary incomplete OR 1.64, 95% CI 1.07 – 2.58; Secondary complete OR 1.75 95% CI 1.05 – 3.21, p = 0.06). We observed weak evidence that distance of clusters further from developed areas associated with referral rates (distance to stable

night lights; p 0.06), with higher referral rates 1–10 km developed areas, which diminished once over 10 km. These findings suggest sociodemographic factors, namely age, of CHWs explains some of the observed variation in referral rates with absolute location less influential.

Cases referred during screening were initially identified by household members selecting photographic case definitions. The pictures were distributed across 12 pages and the total number of times each type of lesion was identified by the individual referral is presented in Appendix Table 3. Multiple lesion types could be selected by an individual referral and in total 3,225 skin lesions were identified among 3,087 referrals with available information. The most common lesion for referred for verification was an enlarged scrotum (23.6% of lesions). Hypopigmented skin patches (16.0%) and BU-like nodules or BU-like plaques (17.6% combined) were also common. Presentations associated with advanced stages of leprosy, deformities of the hands and feet (4.1%) and lepromatous leprosy presentations (7.6%) were the least common reason for referral.

Health districts are arranged left-right in order of south to north geographic location (an approximate proxy for the increasing rural nature of the county along this axis). Table highlights the total households and persons screened and consenting during community screening for skin NTDs alongside referral rates calculate per 100 persons screened by CHWs. District-level referral rates showed statistical evidence of variation after accounting for cluster-level variance (likelihood ratio test p = 0.02).

Disease-Specific Clinical Epidemiology: Buruli Ulcer

During verification of suspected cases, clinical diagnosis was made for suspected Buruli ulcer and laboratory samples (swabs or FNA) were collected from active lesions for confirmation by *IS2404* PCR. The verification teams diagnosed 55 total cases of clinically suspicious BU of which 1 (1.8%) was confirmed by PCR. We identified 3 additional cases of BU through PCR whose initial clinical suspicion was yaws (2) or tropical ulcer (1). All four PCR confirmed cases presented with a single ulcerative form on the lower limb ranging between 2–7cm in diameter. The ages of the confirmed cases were 3, 15, 18 and 50 years old with 50% female. The lesions among two of the cases had begun within the past 12 weeks whereas for the other two cases, symptoms had been present for over 1 and 3 years respectively.

Among clinically suspicious BU cases only, the median age was older (44 years) and 42.0% of the cases were female. Most patients with clinically suspicious lesions reported the persistence of the disease for extensive durations; between 1–3 years (16/55; 29.1%) or over 3 years (26/55, 47.3%). Among these cases 36.4% (20 of 55) reported limitation of movement as a result of the lesion. Most cases had ulcers (39 cases, 70.9%) with 2 instances each of plaque or nodule (3.6% each;). A total of 12 cases were identified with suspected BU osteomyelitis (21.8%). Laboratory confirmation of BU osteomyelitis requires bone collection for confirmation (*3*). We were able to collect clinical material from 7 of 12 actively discharging external lesions all of which were PCR negative. It is plausible that additional BU osteomyelitis cases were within this 12-person cohort. As PCR confirmation is not consistently acquired for BU cases reported to WHO (*4*), we include a sensitivity analysis of prevalence estimates inclusive of clinically suspicious BU cases and excluding 3 PCR confirmed cases. This sensitivity analysis results in a design-adjusted prevalence of 9.8 cases/10,000 persons (95% CI 6.2 – 13.5) for BU and 43.0 per 10,000 for all skin NTDs (95% 36.7 – 49.1).

Disease-Specific Clinical Epidemiology: Leprosy

We diagnosed 39 cases of leprosy during survey activities (4.4 per 10,000; 95% CI 3.3 – 5.5). All patients diagnosed with leprosy were subjected to full-body clinical examination and WHO/ILEP recommended field diagnostic tests (*5*); patch anesthesia testing and assessment of sensory loss in the hands or feet (Appendix Table 4). Patients with suspected lepromatous forms of disease underwent additional examination for bacterial infiltration of the ears or face, and other common lepromatous symptoms (n = 5). Among 39 patients diagnosed with leprosy 21 patients were classified as paucibacillary (53.9%), 16 as multibacillary (41.0%) using WHO case definitions. There were a further 2 cases (5.1%) diagnosed with ongoing leprosy-related complications following completion of treatment. The median age of leprosy patients was 44 (ranged 3 – 87 years old) and 42.8% of cases were female (figure S3). There were 4 cases with missing observations for age and sex. Leprosy cases typically reported the presence of symptoms for over 3 years (25 of 39; 64.1%) although there was a notable proportion of patients who reported the presence of symptoms for less than 1 year (7 of 39; 17.9%). We observed 15 patients with visible leprosy-associated deformities of the hands or feet that were classified as DG2 (38.5%). There were an additional 4 patients classified as DG1 (10.3%) due to sensory loss in the hands or feet without deformity. The remaining 20 patients were diagnosed with leprosy

without any evidence of DG1 or DG2 (51.3%). Despite the high proportion of DG2 cases only 9 of 39 patients (23.1%) reported that the conditions limited their daily activities.

Disease-Specific Clinical Epidemiology: Lymphatic Filariasis–Associated illness

We diagnosed 111 cases of filarial lymphedema (17.5 cases/10,000 persons, 95% CI 14.1–21.0) and 58 cases of filarial hydrocele (8.5 cases/10,000 persons, 95% CI 4.8– 12.3) in Maryland County. All but one case had lymphedema localized to the lower limbs (110 of 111; 99.1%). The remaining case had edema in both the upper arm and lower leg (0.9%). Among all patients the median age was 48 (range 2–86) with 67.3% of cases in females (Appendix Figure 4). One patient <5 years of age was given a diagnosis of filarial lymphedema, which we acknowledge as a probable misclassification.

Verification teams were trained to grade lymphedema according to WHO guidelines (*6*). We observed most grade I cases (59.5%) (Appendix Table 5) followed by the most advanced form, grade III (27.9%), with the fewest observations of grade II (12.6%). Most patients reported being affected by lymphedema for >3 years (88.2%). The proportion of reporting limitation of movement caused by lymphedema was 25.2% but varied between grades (16.7 grade I; 28.6% grade II; 41.9% grade III). One surprising finding was a high proportion of patients diagnosed with bilateral lymphedema (23.4%) (Appendix Table 5). We did not attempt to differentiate between causes of acute pain associated with filarial pathology; acute filarial lymphangitis (AFL) and acute dermatolymphangioadenitis (ADLA) but instead refer to all cases of patient reported pain as ADLA. Nearly all patients reported being affected by ADLA attacks (97.3%) with the majority reporting acute attacks in cycles of approximately 1 month (46.6%) or between one and 3 months (32.4%).

For lymphatic filariasis hydrocele patients, the mean age was 43 (range 1–75), although we acknowledge the 1 year-old boy given a diagnosis of lymphatic filariasis hydrocele as a probable misclassification. There was missing data on age for 3 cases of lymphatic filariasis hydrocele. Most patients (77.6%) reported persistence of the condition for >3 years, yet only a few patients reported that the hydrocele limited their movement (13.8%) (Appendix Table 6). Verification teams probed all hydrocele cases on any pain in the scrotum with 86.2% reporting pain with a typical periodicity of monthly (44.0%) or between 1–3 months (28.0%). Most patients also reported swollen lymph nodes (76.0%) and fever associated with the pain (92.0%).

Disease-Specific Clinical Epidemiology: Yaws

We identified 24 cases of serologically confirmed active yaws in Maryland County (2.6 cases/10,000 persons, 95% CI 1.4 – 3.9). Verification teams were trained to identify both clinically suspected yaws papillomas and ulcers. Patients were initially tested with a rapid treponemal test (SD. Bioline) and if this was positive with a dual treponemal and nontreponemal point of care test (DPP Syphilis Screen and Confirm) In total 34 patients were recorded with clinically suspicious yaws-like lesions of which 22 were positive for treponemal and nontreponemal antibodies (64.7%). Of the remaining 12 clinically suspicious yaws lesion, 2 were positive for treponemal antibody only with the remaining 10 treponemal negative. We also observed 36 persons diagnosed with an ulcer that was not believed clinically to be caused by yaws or BU. Of these persons we observed 2 with dual-positive syphilis serologic results (5.6%), 2 with treponemal antibody response only, with the remaining 32 patients negative for treponemal antibodies. Although the survey case definition was defined serologically, we also tested all yaws lesions for *Treponema pallidum* spp. *pertenue* by using PCR. Among 24 serologically positive cases 17 of 24 lesions (70.8%) were positive for the presence of treponemal DNA. Among 22 serologically confirmed cases, 19 participants were <15 years of age (86.3%) with a median age of 10 years (range 2–32 years) (Appendix Table 7). There was missing data on age for 2 case-patients. A 32 year-old man had the only yaws case diagnosed in persons >18 years of age. Among the 24 case-patients, most diagnoses were made in men (18/24, 75.0%). The primary clinical presentation among yaws cases was evenly distributed between ulcerative and papillomatous forms of disease (50.0%). The duration for which patients reported having active lesions was mostly <1 year (83.3%) but ranged between <8 weeks (37.5%) to >3 years (12.5%).

Spatial Heterogeneity and Coendemicity

To demonstrate the spatial heterogeneity of skin NTDs in Maryland County we present occurrence maps for all skin NTDs (Figure 3 main text) and disease-specific outcome data at both health district and cluster level (Appendix Table 8 and Appendix Figure 5). In Liberia health districts do not follow typical WHO definitions of health district based on population sizes. They instead represent sub-districts by with population sizes range from 8,492 to 51,959. Appendix Table 8 supports Figure 3 of the main text with estimates for all diseases at health district level. For the primary outcome estimates ranged from 14.5 cases/10,000 persons (95% CI

8.4–20.5) in Pleebo to 75.7 cases/10,000 persons (95% CI 59.9– 91.4) in Harper district. Although we did not design the survey with precision to measure differences at health district-level, primary outcome and individual diseases demonstrated overt variation in occurrence and magnitude at these implementation levels. These data also show that at health district level, the predominant pattern is co-endemicity of most skin NTDs although specific diseases, namely yaws and BU, can remain absent. With the low prevalence of BU and yaws, however, we cannot confidently assert that the diseases are absent these implementation levels.

Appendix Figure 5 summarizes the prevalence of each disease by cluster; the smallest survey unit of evaluation (population interquartile range 411–793). At this level, almost three fourths (73.9%; 68/92) of clusters exhibited ≥1 case of any skin NTD. The maximum prevalence observed for combined skin NTD outcomes was 330 cases/10,000 persons (3.3%). For individual diseases, the maximum cluster-level prevalence was 0.4% for BU, 0.7% for leprosy, 2.1% for filarial lymphedema, 2.6% for hydrocele and 2.0% for yaws. Quantifying individual disease patterns at cluster level showed that most clusters were did not have BU (88/92, 95.7%), leprosy (65/92, 70.7%), and yaws (84/92, 91.3%) whereas combined lymphatic filariasis was absent in 43.5% of clusters (40/92). The clustering of disease outcomes is further demonstrated by high ICC values (0.18–0.93) in Table 2 of the main text. Both the disease-specific ICC values and Appendix Figure 5 highlight the predominant pattern of skin NTDs observed at cluster or community level; high prevalence within a limited number of foci with total absence from most of clusters.

With the emergence of integrated skin NTD programs, the scale and structure of disease co-endemicity remains essential. Appendix Figure 6 summarizes the variation in coendemicity of diseases within clusters through intersection plots. Quantifying this difference, across the 92 survey clusters, we identified 9 unique combinations of skin NTD co-occurrence. The most common community-level outcome was lymphatic filariasis only (35 clusters) while single-disease foci were observed for each disease aside from BU (BU 0 clusters; leprosy 10 clusters; yaws 1 cluster). There were 22 of 92 clusters (23.9%) in which two or more skin NTDs were co-endemic, with the leprosy and lymphatic filariasis most commonly found within the same community (24 clusters). Only 1 cluster demonstrated co-occurrence of 3 skin NTDs.

Relative to an alternative approach of vertical survey activities, these data provide strong epidemiologic justification for the efficiency gains made through integration. Using single disease focused estimation strategies, most clusters would have zero reported cases versus an integrated model, under which multiple skin NTDs can be simultaneously identified. Although coendemicity is not the predominant pattern at cluster-level, it is not uncommon. Disease co-occurrence appears to become more predominant at health district level where most skin NTDs coexist.

QC of Screening

During QC of screening we identified a subpopulation of households who reported that they were not visited by CHWs during community screening. To assess the equity of community-based approaches led by CHWs, we attempted to identify socioeconomic indicators that may be associated with nonparticipation (Methods).

Among 1,379 consenting households, we identified 52 households that were not visited by CHWs during screening. We selected 142 matched controls households for final analysis. Univariate analysis indicated that using more expensive cooking fuel (OR 2.86, 95% CI 1.06–8.08), total residents within the household (OR 0.88, 95% CI 0.78–1.01), or crowding (OR 0.46, 95% CI 0.20−1.04), showed some evidence of association with nonparticipation in screening (Appendix Table 9). The independent strength of associations was not evident within the final model (OR 2.33, 95% CI 0.78–6.9; p = 0.13, residents OR 0.94, 95% CI 0.81–1.09; p = 0.40, crowding p = 0.59). These results provide no evidence that socioeconomic status of the household was associated with exclusion from CHW screening. Coupled with high CHW household coverage rates estimated from QC surveys; these findings support the equitable nature of CHW screening for skin NTDs.

Sensitivity Analysis of Prevalence Estimates

QC surveys of CHW screening identified the sensitivity of identifying skin NTD lesions using photo-based screening methods. Using this information, we estimate the effect on survey outcomes through sensitivity analysis by adjusting prevalence rates and their CIs accordingly (*7*). Because our evaluation methods did not enable us to understand variation in sensitivity by absolute location or individual skin NTD outcome, sensitivity analyses are not adjusted to account for these factors. By quantifying the new case detection rate from full body skin

examinations during QC surveys (3.6 cases/100 persons examined; 95% CI 3.1–4.2), we estimated that the total number of referable cases among the survey population was as high as 5,137 (vs. 3,087 reported by CHWs: sensitivity 60.1%). Assuming that skin NTDs are diagnosed at the same rate among these cases, this increased the maximum prevalence across all skin NTDs to 56.5 cases/10,000 persons (95% CI 48.4–64.7) from 34.0 cases/10,000 persons. Appendix Table 10 shows how potential reductions in sensitivity may affect final prevalence estimates for each disease outcome.

References

1. United Nations. World population prospects - population division [2019 Nov 9]. https://population.un.org/wpp

2. Sim J, Wright CC. The kappa statistic in reliability studies: use, interpretation, and sample size requirements. Phys Ther. 2005;85:257–68. PubMed https://doi.org/10.1093/ptj/85.3.257

3. Portaels F, editor. Laboratory diagnosis of Buruli ulcer disease, 2014 [cited 2022 Jul 4]. https://apps.who.int/iris/bitstream/handle/10665/111738/9789241505703_eng.pdf;jsessionid=1283EB242EB7AB6272961A4A7AA2DE57?sequence=AB

4. Omansen TF, Erbowor-Becksen A, Yotsu R, van der Werf TS, Tiendrebeogo A, Grout L, et al. Global epidemiology of Buruli ulcer, 2010–2017, and analysis of 2014 WHO programmatic targets. Emerg Infect Dis. 2019;25:2183–90. PubMed https://doi.org/10.3201/eid2512.190427

5. ILEP. How to diagnose and treat leprosy, 2001 [cited 2022 Jul 4]. https://ilepfederation.org/wp-content/uploads/2020/02/LG1_V2-.pdf

6. Dreyer G, Addiss D, Bettinger J, Dreyer P, Norões J, Rio F. Lymphoedema staff manual: treatment and prevention of problems associated with lymphatic filariasis. Geneva: World Health Organization; 2001.

7. Diggle PJ. Estimating prevalence using an imperfect test. Epidemiol Res Int. 2011;2011:1–5. https://doi.org/10.1155/2011/608719

Appendix Table 1. Referral rates of skin NTDs recorded by CHWs at health district-level across Maryland County, Liberia

Category	Harper	Pleebo	Kaluway #2	Kaluway #1	Barrobo-wojah	Barrobo-fajah
Total households	2,557	3,821	1,400	775	834	620
Total consenting	2,511 (98.2%)	3,775 (98.8%)	1,392 (99.4%)	769 (99.2%)	807 (96.8%)	610 (98.4%)
Individuals screened (% of survey pop)	13,909 (24.5%)	22,712 (40.0%)	8,060 (14.1%)	4,265 (7.5%)	4,659 (8.2%)	3,220 (5.7%)
Number present to see photos	7,998 (57.5%)	13,132 (57.8%)	5,681 (70.5%)	2,791 (65.4%)	3,187 (68.4%)	2,127 (66.1%)
Suspected skin NTDs (% of survey referrals)	938 (31.5%)	1,235 (40.0%)	356 (11.5%)	134 (4.3%)	266 (8.6%)	125 (4.0%)
Referral rate (95% CI)	7.0 per 100 (5.3–8.7)	5.4 per 100 (4.1–6.8)	4.4 per 100 (3.0–5.8)	3.1 per 100 (2.5–3.8)	5.7 per 100 (3.1–8.3)	3.9 per 100 (2.1–5.7)

Appendix Table 2. Implementation factors associated with probability of referral for a suspected skin NTD lesion by community health workers

Exposure	Referred households (n/N)	OR (95% CI)	p-value	Adjusted OR (95% CI)	p-value*
Household-level factors					
# household members†	-	1.08 (1.06 – 1.10)	<0.0001	1.09 (1.07–1.11)	<0.0001
Proportion male†	-	1.60 (1.27 – 2.01)	<0.0001	1.61 (1.28–2.03)	<0.0001
Proportion present to view photos	-	1.47 (1.19 – 1.81)	0.0003	1.46 (1.18–1.81)	0.0005
Crowding (persons per room)	-	1.00 (0.92 – 1.09)	0.98	0.98 (0.90–1.07)	0.60
Cluster-level environmental factors					
Distance to stable night lights†					
1km or less	629/3,036	1		1	
>1km – 10km	955/3,417	1.19 (0.60 – 2.36)	0.23	1.32 (0.64–2.72)	0.06
>10km	727/3,401	0.86 (0.44 – 1.69)		0.85 (0.42–1.68)	
Urbanization†					
Rural	1,890/7,741	1	0.62	1	0.16
Peri-urban or urban	421/2,113	0.83 (0.39 – 1.76)		0.59 (0.31–1.21)	
Cluster-level CHW factors Age of CHW					
35 or under	1,280/4,777	1	<0.0001	1	0.001
Over 35	1,031/5,077	0.52 (0.38 – 0.70)		0.59 (0.43–0.81)	
Sex of CHW					
Male	1,692/7,220	1	0.67	1	0.40
Female	619/2,634	1.09 (0.72 – 1.62)		1.17 (0.80–1.70)	
CHW training level					
CHV	1,566/7,174	1	0.09	1	0.34
CHA	745/2,680	1.33 (0.96 – 1.85)		1.19 (0.83–1.70)	
Education of CHW					
Primary or below	133/884	1		1	
Secondary incomplete	1,269/5,004	1.90 (1.25 – 2.87)	0.006	1.64 (1.06–2.57)	0.08
Secondary complete	909/3,966	2.21 (1.27 – 3.84)		1.75 (1.00–3.08)	
Years working as CHW	-	0.95 (0.91 – 0.99)	0.01	0.97 (0.93–1.01)	0.19
CHW phone ownership					
No	516/2,141	1	0.49	1	0.65
Yes	1,796/7,713	1.14 (0.78 – 1.67)		1.08 (0.76–1.53)	

*P-values calculated by likelihood ratio test. 5 of a total sample of 9,859 households were not included due to missing observations.
†Indicates the predictors included in baseline OR model.

Appendix Table 3. Types of skin lesions identified by household members during CHW screening as the reason for referral*

Photos selected							
Ulcer	Nodule or plaque	Papilloma	Hypopigmented patch	Deformity of hand or foot	Lepromatous Leprosy	Limb edema	Enlarged scrotum
309 (9.6%)	569 (17.6%)	415 (12.9%)	515 (16.0%)	133 (4.1%)	232 (7.2%)	292 (9.1%)	760 (23.6%)

*The left to right order of the table represents the order in which the pictures were shown by CHWs using the flipbooks. There was missing data on referral photo for 101 individuals (n = 3,087).

Appendix Table 4. Clinical characteristics of all patients diagnosed with leprosy

Clinical characteristic	No.. (%)
Categorization	
Paucibacillary	21 (53.9%)
Multibacillary	16 (41.0%)
Treatment complete with complication	2 (5.1%)
Limitation of daily activities	
No	30
Yes	9 (23.1%)
Duration of lesions	
Less than 1 y	7 (17.9%)
1–3 y	4 (10.3%)
Over 3 y	25 (64.1%)
Unknown	3 (7.7%)
Deformity of hands or feet (DG2)	
No	24
Yes	15 (38.5%)
Patch anesthesia	
No	20
Yes	19 (48.7%)
Sensory loss in hands or feet	
No	25
Yes	14 (35.9%)
Nerve enlargement	
No	26
Yes	13 (33.3%)
Deformity or sensory loss (DG1 or DG2)	
No	20
Yes	19 (48.7%)

Appendix Table 5. Clinical characteristics of all patients diagnosed with filarial lymphedema

Category	Grade 1	Grade 2	Grade 3	Total
Anatomic location				
Lower limb and upper limb				1 (0.9%)
Lower limb				110 (99.1%)
LF-lymphedema categorization	66 (59.5%, 95% CI 49.7–68.7)	14 (12.6%, 95% CI 7.1 – 20.3)	31 (27.9%, 95% CI 19.8 – 37.2)	
Limitation of movement				
No	55	10	18	83 (74.8%)
Yes	11 (16.7%)	4 (28.6%)	13 (41.9%)	28 (25.2%)
Known duration of lymphedema				
Less than 1 y	2 (3.0%)	1 (7.1%)	1 (3.2%)	4 (3.6%)
1–3 y	6 (9.1%)	1 (7.1%)	0	7 (6.3%)
Over 3 y	58 (87.9%)	11 (78.6%)	29 (93.5%)	98 (88.2%)
Unknown	0	1 (7.1%)	1 (3.2%)	2 (1.8%)
Reversible swelling				
No	4 (6.1%)	10 (71.4%)	28 (90.3%)	42 (37.8%)
Yes	61 (92.4%)	4 (28.6%)	3 (9.7%)	68 (61.3%)
Unknown	1 (1.5%)	0	0	1 (0.9%)
Pitting edema				
No	38	1	1	40 (36.0%)
Yes	28 (42.4%)	13 (92.9%)	30 (96.8%)	71 (64.0%)
Unilateral vs bilateral lymphedema				
Unilateral	54	10	21	85 (76.6%)
Bilateral	12 (18.2%)	4 (28.6%)	10 (32.3)	26 (23.4%)
History of heart disease				
No	57 (86.4%)	14 (100%)	28 (90.3%)	99 (89.2%)
Yes	5 (7.6%)	0	1 (3.2%)	6 (5.4%)
Unknown	4 (6.0%)	0	2 (6.5%)	6 (5.4%)
Hard or thickened skin on affected limb				
No	35	2	2	39 (35.1%)
Yes	31 (47.0%)	12 (85.7%)	29 (93.5%)	72 (64.9%)
Skin folds				
No	52	6	1	59 (53.2%)
Yes	14 (21.2%)	8 (57.1%)	30 (96.8%)	25 (46.9%)
Acute attacks of pain in affected limb (ADLA)				

Category	Grade 1	Grade 2	Grade 3	Total
No	3	0	0	3 (2.7%)
Yes	63 (95.5%)	14 (100%)	31 (100%)	108 (97.3%)
Periodicity of ADLA				
Daily	1 (1.6%)	1 (7.1%)	0	2(1.9%)
Weekly	1 (1.6%)	1 (7.1%)	2 (6.5%)	4 (3.7%)
Monthly	30 (47.6%)	3 (21.4%)	19 (61.3%)	52 (48.2%)
Every 1–3 mo	23 (36.5%)	7 (50.0%)	6 (19.4%)	36 (33.3%)
Every 6 mo	4 (6.3%)	2 (14.3%)	3 (9.7%)	9 (8.3%)
Annual or greater	1 (1.6%)	0	1 (3.2%)	2 (1.9%)
Unknown	3 (4.8%)	0	0	3 (2.8%)
Swollen lymph nodes during ADLA				
No	7 (11.1%)	1 (7.1%)	4 (12.9%)	12 (11.1%)
Yes	55 (87.3%)	13 (92.9%)	27 (87.1%)	95 (88.0%)
Unknown	1 (1.6%)	0	0	(0.9%)

Appendix Table 6. Clinical characteristics of all patients diagnosed with filarial hydrocele

Clinical characteristic	No. (%)
Limitation of movement	
No	50 (86.2%)
Yes	8 (13.8%)
Duration of hydrocele	
Less than 1 y	3 (5.2%)
1–3 y	8 (13.8%)
Over 3 y	45 (77.6%)
Unknown	2 (3.4%)
Trans-illumination positive	
No	5 (8.6%)
Yes	53 (91.4%)
Acute pain within swollen area	
No	8 (13.8%)
Yes	50 (86.2%)
Periodicity of acute pain	
Daily	3 (6.0%)
Weekly	3 (6.0%)
Monthly	22 (44.0%)
Every 1–3 mo	14 (28.0%)
Every 6 mo	1 (2.0%)
Annual or more	3 (6.0%)
Unknown	4 (8.0%)
Swollen lymph nodes with acute pain	
No	10 (20.0%)
Yes	38 (76.0%)
Unknown	2 (4.0%)
Fever associated with acute pain	
No	4 (8.0%)
Yes	46 (92.0%)

Appendix Table 7. Clinical characteristics of all patients diagnosed with yaws

Clinical characteristic	No. (%)
Yaws cases	
Total DPP positive cases	24
Total PCR positive	17 (70.8%)
Mean age	11.3 (2 - 32)
Proportion of clinically suspicious yaws cases serologically confirmed as yaws	
Treponemal negative	10 (29.4%)
Treponemal positive	2 (5.9%)
Dual positive	22 (64.7%)
Proportion of non-clinically suspicious ulcers serologically confirmed as yaws	
Dual negative	2 (5.6%)
Treponemal positive	2 (5.6%)
Dual positive	32 (88.9%)
Lesion presentation	
Ulcer	12 (50.0%)
Papilloma	12 (50.0%)
Limitation of movement	
No	22 (91.7%)
Yes	2 (8.3%)
Duration of lesion	
Less than 8 weeks	9 (37.5%)
8–26 weeks	6 (25.0%)
27 weeks - 1 y	5 (20.8%)
1–3 y	0
Over 3 y	3 (12.5%)
Unknown	1 (4.2%)

Appendix Table 8. District level estimates of all skin NTD outcomes*

	Prevalence per 10,000 (95% CI)					
Location	All skin NTD	Buruli ulcer	Leprosy	LF lympho.	LF hydrocele	Yaws
Harper	75.6 (59.9 – 91.4)	0.5 (0 – 1.1)	4.2 (2.8 – 5.6)	50.7 (37.5 – 64.0)	20.2 (7.2 – 33.2)	0
Pleebo	14.5 (8.4 – 20.5)	1.1 (0 – 3.0)	2.3 (0.4 – 4.3)	7.2 (3.0 – 11.5)	3.7 (1.4 – 5.9)	0.2 (0.1 – 0.2)
Kaluway 2	33.0 (18.4 – 47.6)	0	8.9 (5.8 – 12.0)	1.0 (0 – 2.2)	2.4 (0.6 – 4.2)	20.7 (8.8 - 32.6)
Kaluway 1	15.0 (4.5 – 25.4)	0	3.0 (0 – 6.8)	3.0 (0 – 6.8)	9.0 (4.2 – 13.8)	0
Barrobo-wojah	34.1 (27.2 – 40.9)	3.5 (1.2 – 5.9)	8.5 (3.2 – 13.9)	4.3 (1.8 – 6.8)	12.1 (8.0 – 16.1)	5.7 (4.5 – 6.9)
Barrobo-fajah	21.7 (-)	0	21.7 (-)	0	0	0

*Prevalence estimates per 10,000 population with 95% confidence intervals. No confidence intervals are provided for Barrobo-fajah as the total population was included within the survey sample.

Appendix Table 9. Household-level factors associated with exclusion from CHW screening

Risk factor	CHW not visited (%)	CHW visited (%)	Baseline conditional OR	p-value	Full model conditional OR	p-value
Number slept last night	-	-	0.88 (0.78 – 1.01)	0.05	0.94 (0.81 – 1.09)	0.40
Proportion male	-	-	0.77 (0.38 – 7.64)	0.49	-	
Crowding						
1 or fewer per room	19 (33.3%)	38 (66.7%)	1	0.16	1	0.59
>1 and <3 per room	22 (22.9%)	74 (77.1%)	0.46 (0.20 – 1.04)		0.62 (0.25 – 1.66)	
3+ per room	11 (26.8%)	30 (73.2%)	0.46 (0.15 – 1.35)		0.60 (0.17 – 2.1)	
Education of household head*						
No education	12 (18.8%)	52 (81.3%)	1	0.42	-	-
Primary	12 (27.9%)	31 (72.1%)	1.76 (0.68 – 4.54)			
Secondary	16 (28.1%)	41 (71.9%)	1.53 (0.66 – 3.56)			
Higher	11 (37.9%)	18 (62.1%)	2.23 (0.78 – 6.35)			
Radio ownership						
No	34 (27.2%)	91 (72.8%)	1	0.78	-	-
Yes	18 (26.1%)	51 (73.9%)	0.90 (0.45 – 1.84)			
Floor material of household						
Natural	26 (26.3%)	73 (73.7%)	1	0.77	-	-
Cement	26 (27.4%)	69 (72.6%)	0.89 (0.40 – 1.99)			
Cooking material of household						
Wood	27 (22.0%)	96 (78.0%)	1	0.04	1	0.13
Charcoal	25 (35.2%)	46 (64.8%)	2.86 (1.06 – 8.08)		2.33 (0.78 – 6.95)	
Bench ownership						
No	3 (5.8%)	6 (4.2%)	1	0.24	-	-
Yes	49 (94.2%)	136 (95.8%)	0.29 (0.03–2.18)			
Latrine ownership						
No	17 (24.6%)	52 (75.4%)		0..94	-	-
Yes	35 (28.0%)	90 (72.0%)	0.97 (0.41 – 2.27)			

*One observation missing data for household head education level.

Appendix Table 10. Sensitivity analysis of total skin NTD cases and prevalence estimates (per 10,000)

Cases	Survey cases, Estimated prevalence per 10,000 (95% CI)	Sensitivity analysis cases, Estimated prevalence per 10,000 (95% CI)
All skin NTD	236, 34.0 (29.1 – 38.9)	393, 56.5 (48.4 – 64.7)
Buruli ulcer	4, 0.9 (0 –1.8)	7, 1.4 (0 – 3.1)
Leprosy	39, 4.4 (3.3 – 5.5)	65, 7.3 (5.4 – 9.2)
LF lymphedema	111, 17.5 (14.1 – 21.0)	185, 29.2 (23.4 – 35.0)
LF hyrdocele	58, 8.5 (4.8 – 12.3)	97, 14.2 (8.0 – 20.4)
Yaws	24, 2.6 (1.4 – 3.9)	40, 4.4 (2.4 – 6.4)

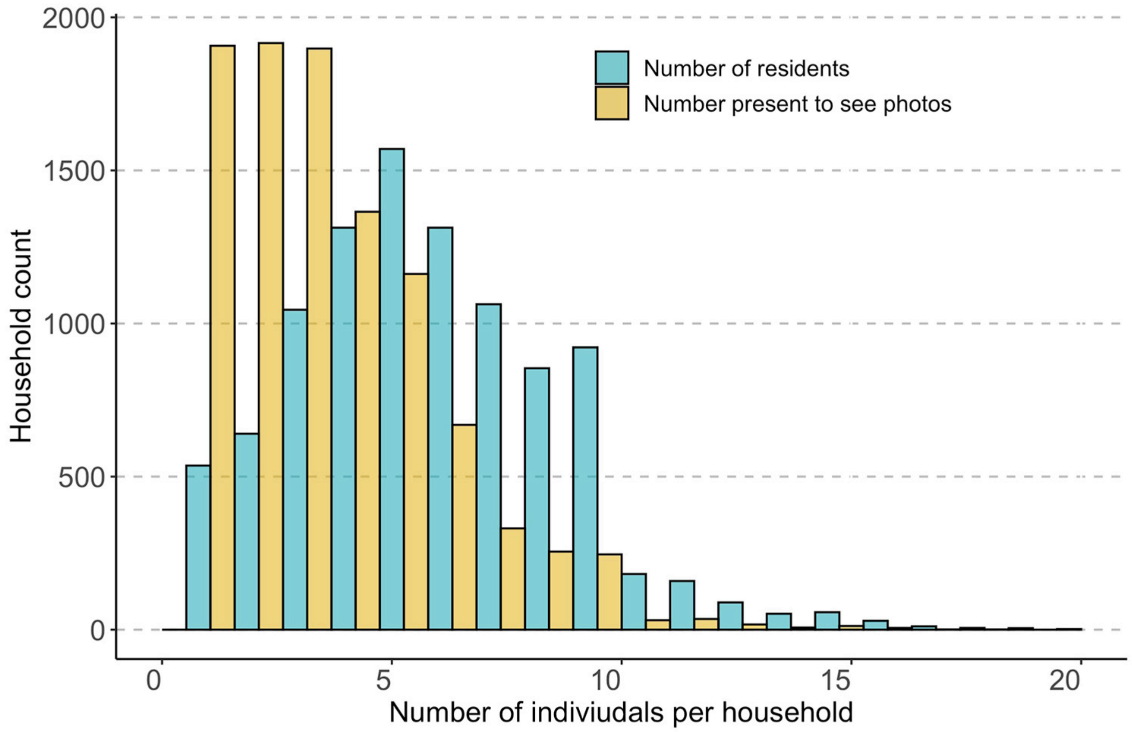

Appendix Figure 1. Number of residents enumerated per household against number of residents present to see photos of skin NTDs during CHW screening. A total of 38.6% of the resident population were absent at the time of CHW visits.

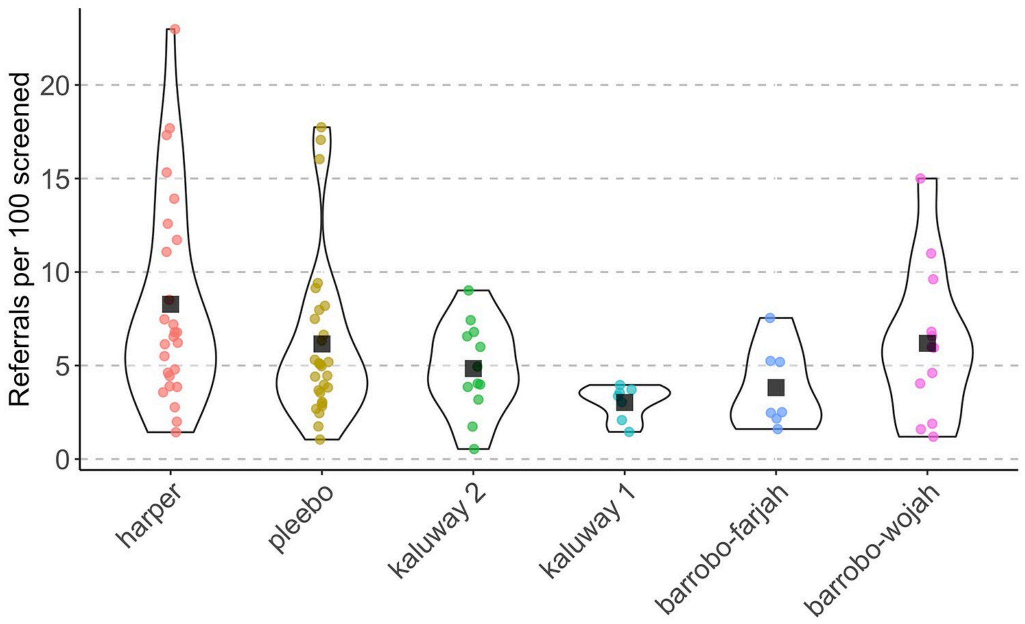

Appendix Figure 2. Violin plot of referral rates of suspected skin NTDs by CHWs at cluster-level (n = 92) visualized by health district. Points represent individual cluster referral rates and squares show mean referral rates for the health district.

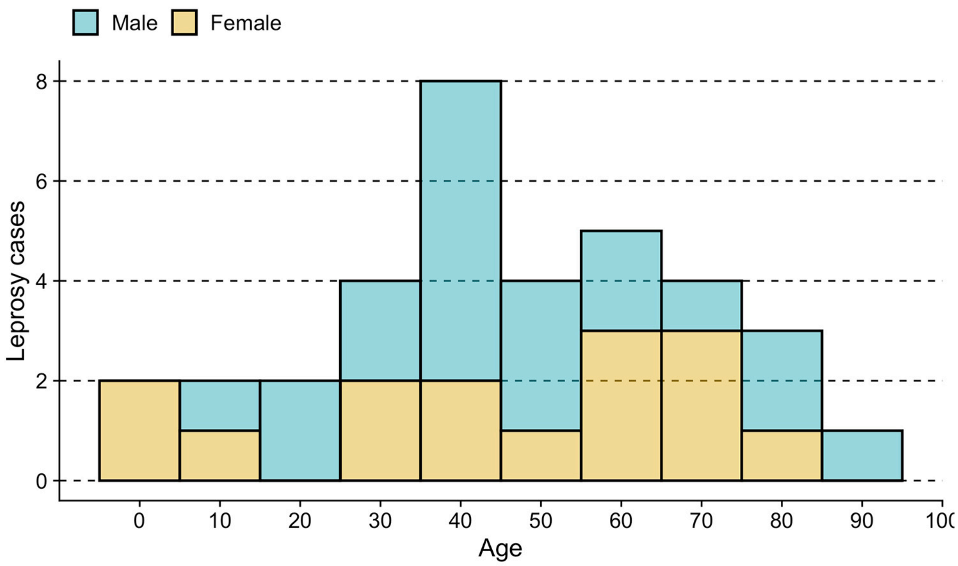

Appendix Figure 3. Age-sex distribution of leprosy cases.

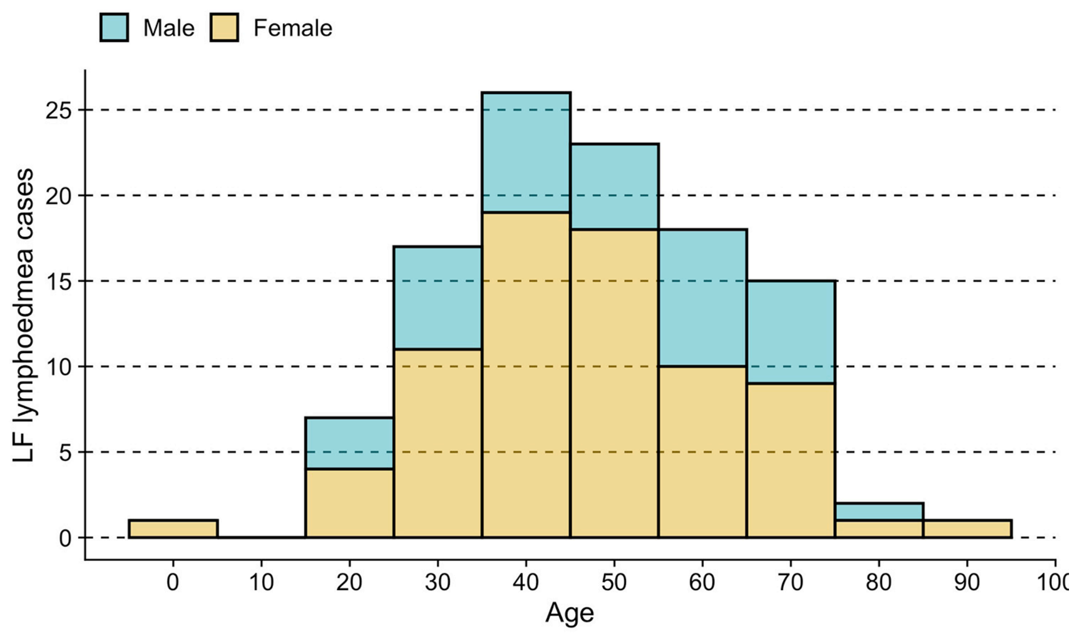

Appendix Figure 4. Age and sex distribution of filarial lymphedema cases.

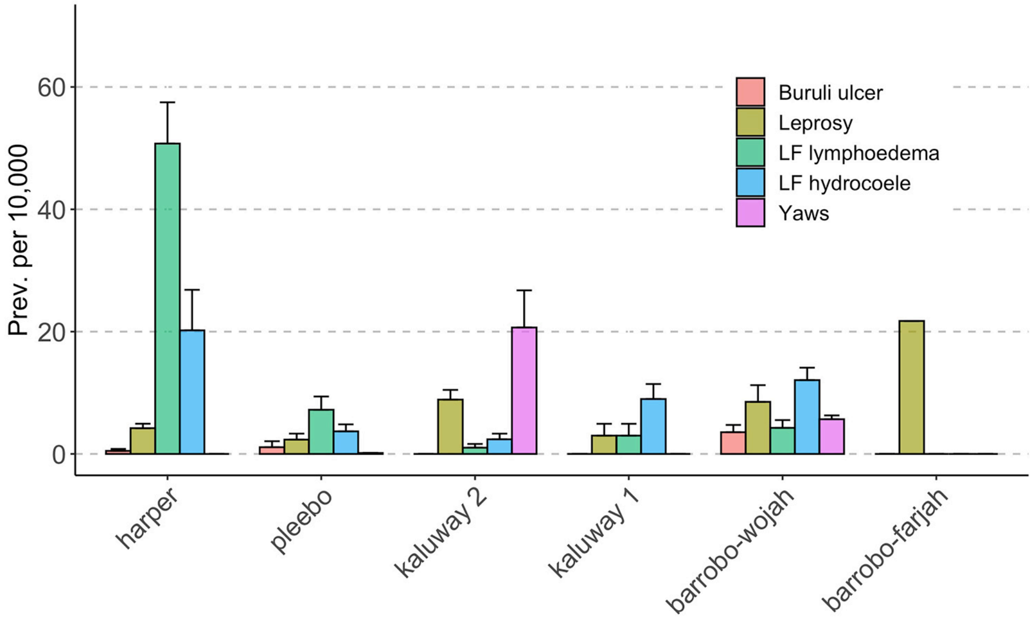

Appendix Figure 5. Age and sex distribution of filarial lymphedema cases. Error bars represent standard error.

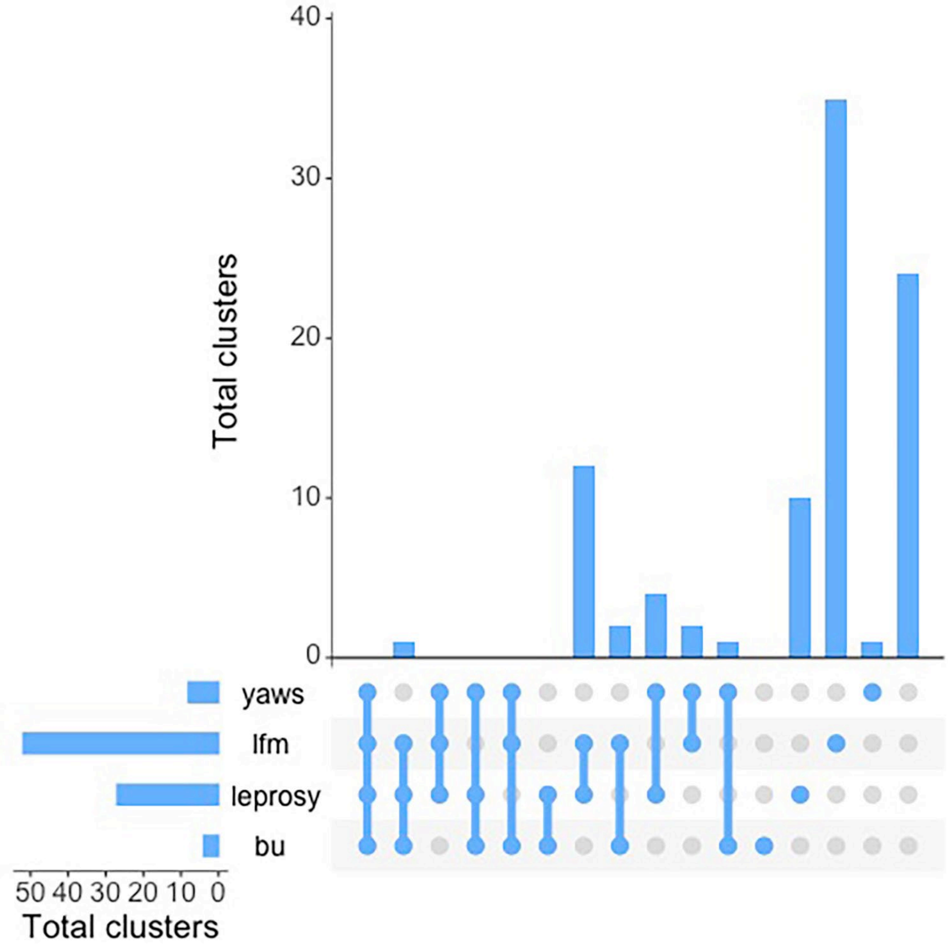

Appendix Figure 6. Cluster co-occurrence plot. Intersection plots highlight the unique co-occurrence outcome of all 92 survey clusters indicated by the outcome matrix (lower panel). The upper panel also highlights the total number of clusters each outcome was identified within.
